# Clinicopathological Significances of Peritumoral Budding in Colorectal Cancer: A Detailed Analysis Based on Mucinous and Micropapillary Pattern

**DOI:** 10.3390/diagnostics13233564

**Published:** 2023-11-29

**Authors:** Jung-Soo Pyo, Nae Yu Kim, Kyueng-Whan Min, Dong-Wook Kang

**Affiliations:** 1 Department of Pathology, Uijeongbu Eulji Medical Center, Eulji University School of Medicine, Uijeongbu-si 11759, Republic of Korea; jspyo@eulji.ac.kr (J.-S.P.); kyueng@eulji.ac.kr (K.-W.M.); 2 Department of Internal Medicine, Uijeongbu Eulji Medical Center, Eulji University School of Medicine, Uijeongbu-si 11759, Republic of Korea; naeyu46@eulji.ac.kr; 3 Department of Pathology, Chungnam National University Sejong Hospital, 20 Bodeum 7-ro, Sejong-si 30099, Republic of Korea; 4 Department of Pathology, Chungnam National University School of Medicine, 266 Munhwa Street, Daejeon 35015, Republic of Korea

**Keywords:** colorectal cancer, peritumoral budding, mucinous component, micropapillary pattern, prognosis

## Abstract

The present study aimed to evaluate the correlations between peritumoral tumor budding (PTB) and the clinicopathological characteristics of colorectal cancer (CRC) according to histological components. The PTBs were investigated and divided into high and low groups. The clinicopathological significance and prognostic implications of PTB in CRC were evaluated. High PTB was found in 104 of 266 CRCs (39.1%). High PTB was significantly correlated with left-sided tumors, lymphatic invasion, lymph node metastasis, distant metastasis, and high pTNM stage. However, there was no significant correlation between PTB and the other clinicopathological characteristics. PTB was significantly higher in CRCs without the mucinous component than those with the mucinous component (*p* = 0.008). However, there was no significant difference between CRCs with and without the micropapillary pattern (*p* = 0.123). Patients with high PTB had worse recurrence-free survival than those with low PTB (*p* = 0.031). In the subgroup analysis based on histological components, a significant correlation between PTB and recurrence-free survival was found in CRC with a micropapillary pattern but not in those without a micropapillary pattern (*p* = 0.010 and *p* = 0.178, respectively). These findings indicate that high PTB is significantly correlated with aggressive tumor behaviors and worse survival in patients with CRC. However, the prognostic implications of PTB can differ according to histological components.

## 1. Introduction

The tumor–node–metastasis (TNM) classification system is a widely–used staging system for malignant tumors, including colorectal cancer (CRC) [1]. This classification system is useful for predicting the prognosis of CRC patients. However, patients with the same stage show a wide range of tumor behaviors and prognoses. Accurate predictions for each patient require a variety of approaches, not just a single predictive tool. Attempts and applications using various evaluation factors have been made to predict the prognoses of CRC patients. Tumor budding (TB) is an important histological feature of the tumor microenvironment. TB is defined as a single tumor cell or a cluster of up to four tumor cells, and is divided into intratumoral TB (ITB) and peritumoral TB (PTB). According to recommendations from the International Tumor Budding Consensus Conference (ITBCC), PTB is evaluated at the invasive front [2]. The grading was recommended by assessing the number of PTBs in a hotspot. According to recommendations from the ITBCC, the three-tiered system is classified into low (0–4), intermediate (5–9), and high grades (≥10) [2]. However, this evaluating system is not fully understood. Unlike PTB, ITB refers to TB within the main tumor body and can be evaluated even in biopsy specimens [2,3]. However, PTB at the invasive front can only be assessed in endoscopic or surgically resected CRCs. TB has been reported to be a poor prognostic factor in CRC [4,5,6,7,8]. In addition, TB is significantly correlated with aggressive tumor behaviors, such as lymphovascular invasion and lymph node and distant metastases [4,5,6,7,8]. In Stage II CRCs, high-grade TB is suspected to result in a worse prognosis and may be considered for adjuvant therapy [6,7,9,10]. However, depending on the tumor stage of the CRC, the prognostic implication of the intermediate grade may be different [2].

CRCs are classified into various histological subtypes, including micropapillary and mucinous carcinomas. Micropapillary carcinoma is a rare histological subtype in CRCs [11]. A micropapillary pattern is frequently found in up to 19.1% of CRCs [11]. It is well–known that micropapillary carcinoma has frequent lymphovascular invasion and lymph node metastasis [12]. The presence of a micropapillary pattern was significantly correlated with a higher pT stage, which is inconsistent with previous studies [11]. In addition, there was a significant correlation between micropapillary pattern and worse prognosis, regardless of the proportion of micropapillary pattern in CRCs [11]. However, the clinicopathological significances of the micropapillary pattern may differ according to previous reports. Micropapillary patterns, defined as small cell clusters in lacunar spaces, can be confused with lymphovascular invasion and TB [11]. However, the clinicopathological significance of TB is not fully understood in CRCs with a micropapillary pattern.

Mucinous carcinoma, by definition, includes a substantial amount of mucin [13]. The tumor cells of mucinous carcinoma are located in the extracellular mucin. Mucinous carcinomas were frequently found in the right colon and were associated with high frequency of microsatellite instability [14]. The prognostic impacts of mucinous carcinomas are not consistent between studies [14,15,16,17]. In addition, the prognosis of mucinous carcinomas may differ across different tumor locations [14]. However, the correlation between TB and mucinous carcinoma in CRCs remains unclear. Here, we aimed to elucidate the clinicopathological significance of PTB in CRCs. The correlations between PTB and the clinicopathological characteristics of CRC were investigated in detail. The distribution of PTB, such as mucinous and micropapillary components, was investigated according to histological components. In addition, the prognostic impact of PTB was evaluated based on histological components.

## 2. Materials and Methods

### 2.1. Patients and Specimens

The files of 266 patients who had undergone surgical resection of CRCs in the Eulji University Medical Center, Eulji University School of Medicine (Republic of Korea) from 1 January 2001 to 31 December 2010 were retrospectively analyzed. We reviewed the medical charts, pathological records, and glass slides to obtain information on clinicopathological characteristics, including PTB, age, sex, tumor size, location, differentiation, invasion depth, lymphovascular invasion, perineural invasion, lymph node metastasis, metastatic lymph node ratio, distant metastasis, and pathologic TNM (pTNM) stages. All histological features were evaluated by referring to the *AJCC Cancer Staging Manual* [1]. The tumor location was divided into the right and left colon. Rectal cancers were included in the left colon. The metastatic lymph node ratio is defined as the ratio of the number of metastatic lymph nodes to the number of examined lymph nodes. The pathologic tumor (pT) stage was divided into pT1-2 and pT3-4 groups. In addition, the pTNM stage was divided into pTNM I-II and III-IV groups. This protocol was reviewed and approved by the Institutional Review Board of Uijeongbu Eulji University Hospital (Approval No. UEMC 2023-07-004).

### 2.2. Evaluation of Peritumoral Budding

A single tumor cell or cluster of up to four tumor cells at the invasive front are considered to be PTB. In the present study, PTB was investigated at the tumor’s invasive front. The number of PTB was counted in a hotspot (area = 0.785 mm^2^). To evaluate the clinicopathological and prognostic significances of PTB, the PTB groups and clinicopathological characteristics and prognosis were compared. In the present study, the PTB groups were divided into high and low groups according to the number of PTBs. As a criterion for dividing the high and low groups, 10 PTBs per hotspot (area = 0.785 mm^2^) at the invasive front were used; the low PTB had fewer than 10 PTBs per hotspot, and the high PTB had 10 or more PTBs per hotspot. 

### 2.3. Evaluation of Mucinous and Micropapillary Components

The presence of mucinous and micropapillary components was investigated to evaluate the correlation between PTB and histologic components. The micropapillary pattern has the following characteristics: (1) tumor cell clusters without fibrovascular cores; (2) tumor cells with pleomorphic nuclei and eosinophilic cytoplasm; (3) tumor cells with a reverse nuclear polarity; and (4) tumor cells located in the stromal spaces [11]. In CRC with mucinous component, tumor cells float in the extracellular mucin. By definition, the mucinous adenocarcinoma includes the mucin pools in >50% of the tumor. However, in the present study, mucinous adenocarcinoma was not included. The presence of mucinous and micropapillary components was evaluated regardless of the ratio. In 25 cases, tumor cells with micropapillary patterns were identified in mucin pools. These cases were also included in the group with micropapillary component. 

### 2.4. Statistical Analysis

Statistical analyses were performed using the SPSS version 22.0 software (SPSS, Chicago, IL, USA). The significance of the correlation between PTB and the clinicopathological characteristics, including sex, tumor size, location of tumor, tumor differentiation, vascular, lymphatic, and perineural invasions, pathologic tumor (pT) stage, lymph node metastasis, and distant metastasis, was determined using either the χ^2^ test or Fisher’s exact test (two-sided). Comparisons between PTB and age, tumor size, and metastatic lymph node ratio were analyzed using a two-tailed Student’s *t*-test. In addition, the correlations between PTB and histological components, including the presence of mucinous component and micropapillary pattern, were evaluated using either the χ^2^ test or Fisher’s exact test (two-sided). Survival curves were estimated using the Kaplan–Meier product-limit method, and differences between the survival curves were determined to be significant on the basis of the log-rank test. In addition, we performed the multivariate Cox regression analysis to determine if PTB is an independent prognostic marker when other known risk factors are adjusted for. The results were considered statistically significant at *p* < 0.05.

## 3. Results

### 3.1. The Correlation between Peritumoral Budding and Clinicopathological Characteristics

**Figure 1:**
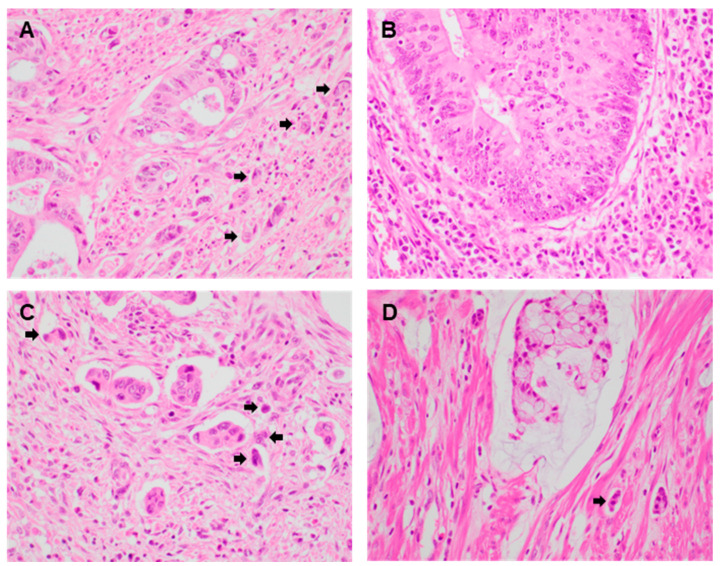
Representative images show colorectal cancers with peritumoral budding ((**A**–**D**); Arrows: peritumoral budding). (**A**) Conventional colorectal adenocarcinoma with high peritumoral budding (×400). (**B**) Conventional colorectal adenocarcinoma with low peritumoral budding (×400). (**C**) Colorectal adenocarcinoma with micropapillary pattern (×400). (**D**) Colorectal adenocarcinoma with mucinous component (×400).

Representative PTB images are shown in Figure 1. All images are obtained from a microscope at 200× magnification. Figure 1A,B showed the representative images as high and low PTB groups. Figure 1C,D showed the representative images as CRCs with micropapillary and mucinous components. In the present study, which is based on ten PTBs, patients with CRC were divided into high- and low-PTB groups. High PTB was found in 104 of 266 CRCs (39.1%) (Table 1). PTB was more frequently identified in left-sided than right-sided tumors (*p* = 0.024). The presence of PTB was significantly correlated with lymphatic invasion and lymph node metastases (*p* = 0.033 and *p* = 0.016, respectively). In addition, there were significant correlations between the presence of PTB and distant metastases as well as higher pTNM stages (*p* = 0.009 and *p* = 0.016, respectively). However, there were no significant differences in other clinicopathological characteristics between CRCs with high and low PTB. High PTB rates were not significantly different in male and female patients (*p* = 0.707). The tumor sizes in high- and low-PTB groups were 5.57 ± 1.85 cm and 5.39 ± 2.21 cm, respectively (*p* = 0.505). The rate of high PTB was slightly higher in poorly differentiated tumors than in well or moderately differentiated tumors (*p* = 0.282). There was no significant difference of vascular and perineural invasion between the high- and low-PTB groups (*p* = 0.515 and *p* = 0.063, respectively). In addition, there was no difference between PTB and pT stages (*p* = 0.602). Metastatic lymph node ratios were 0.16 ± 0.24 and 0.11 ± 0.21 in high-PTB groups, respectively (*p* = 0.087). However, there was no significant correlation between PTB and distant metastatic site (*p* = 0.355)

Next, the correlations between PTB and histological components were evaluated. In 266 CRCs, mucinous and micropapillary components were identified in 46 (17.3%) and 74 (27.8%) cases, respectively. CRCs with a mucinous component were significantly correlated with low PTB (*p* = 0.008; Table 2). In CRCs with no mucinous component (*n* = 220), high and low PTB cases were 94 and 126 cases, respectively. In CRCs with mucinous component, high PTB was less frequently found. However, there was no significant correlation between PTB and the micropapillary pattern (*p* = 0.123). High PTB rates were 31.1% (23/74) and 42.2% (81/192) in CRCs with and without a micropapillary component, respectively.

### 3.2. The Correlation between Peritumoral Budding and Survival

**Figure 2:**
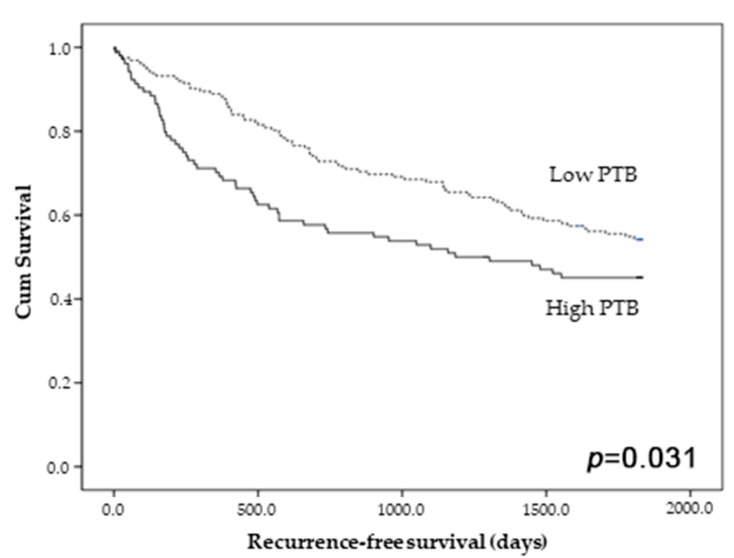
Recurrence-free survival according to the presence of peritumoral budding (PTB) in overall colorectal cancers. *p* < 0.05 is highlighted in Italic bold.

**Figure 3:**
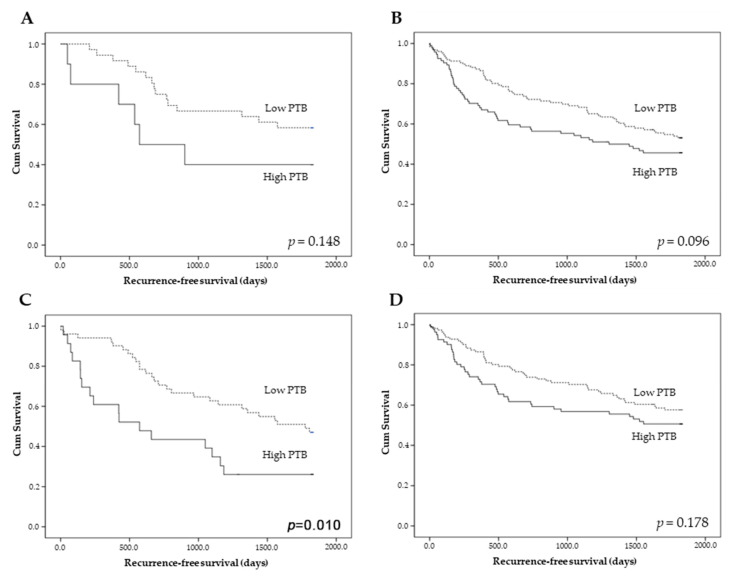
Recurrence-free survival according to the presence of peritumoral budding (PTB) in colorectal cancers (**A**–**D**). (**A**) Adenocarcinoma with a mucinous component. (**B**) Adenocarcinoma without a mucinous component (**C**) Adenocarcinoma with a micropapillary pattern. (**D**) Adenocarcinoma without a micropapillary pattern. *p* < 0.05 is highlighted in Italic bold.

The prognostic impact of PTB was evaluated in overall cases. CRCs with high PTB had worse recurrence-free survival than those with low PTB (*p* = 0.031; Figure 2). A detailed analysis based on the presence of histological components, such as mucinous and micropapillary components, was performed. The presence of a mucinous component had no prognostic impact on the prognostic stratification between the high- and low-PTB groups (Figure 3A,B). However, among the CRCs with a micropapillary component, those with high PTB showed worse recurrence-free survival than those with low PTB (*p* = 0.010; Figure 3C). There was no significant difference in recurrence-free survival between the high- and low-PTB subgroups in CRCs without a micropapillary component (*p* = 0.178; Figure 3D). In multivariate analysis, high PTB was an independent predictor of worse recurrence-free survival (*p* = 0.047, hazard ratio 1.464, 95% confidence interval 1.005–2.133).

## 4. Discussion

PTB is defined as a single tumor or cluster of up to four tumor cells at the invasive front [2]. PTB is associated with an aggressive clinical course in CRC [2,5,18,19,20]. The present study aimed to investigate the correlation between PTB and the histological components of CRCs. The results of our study were as follows: (1) PTB was significantly correlated with aggressive tumor behaviors, such as lymphatic invasion, lymph node metastasis, and distant metastasis. (2) PTB was significantly higher in CRCs without a mucinous component than those with a mucinous component. (3) There was no significant correlation between PTB and the micropapillary component. (4) CRCs with high PTB had a worse prognosis than those with low PTB. (5) The prognostic impact of PTB differed with the histological CRC component’s presence. The present study is, to the best of our knowledge, the first to compare the impact of PTB in CRCs with micropapillary and mucinous components.

The ITBCC and the College of American Pathologists (CAP) proposed a method for evaluating and grading PTB through a hotspot [2]. In our study, we also identified hotspots and evaluated PTB at the invasive front. In the ITBCC guidelines, the area of one hotspot is 0.785 mm^2^ [2]. In addition, the hotspot method is recommended [2]. These statements are strong recommendations with a moderate quality of evidence [2]. Roger et al. reported the impact of TB through a systematic review and meta-analysis [8], and the results of the meta-analysis provided the evidence for the ITBCC guidelines [2,8]. This meta-analysis included 34 eligible studies [8]. The eligible studies used various evaluation methods for PTBs [8]. Regardless of evaluation methods, PTB was significantly correlated with lymph node metastasis and recurrence [8]. Some researchers have chosen to survey areas for PTB by counting the number of PTB in 10 consecutive HPFs [21]. This is similar to the evaluation of HER2 expression and mitosis in breast cancer. It is recommended that PTB be assessed at the hotspot that has the highest number of PTBs [2]. Although this is more convenient than evaluating across 10 HPFs, it can be challenging to find a hotspot and count PTBs. In daily practice, representative sections are obtained from the tumor; therefore, sections of the entire tumor are not evaluated for PTB. This raises the question of whether the microscopic assessment of PTB is the most representative. Finding a hotspot relies entirely on a pathologist; therefore, exploring more fields, such as 10 HPFs, is also necessary. Karamitopoulou et al. reported the usefulness of the 10 HPF scoring method for evaluating PTB in CRCs [22]. The method of observing multiple fields has been reported to be more representative and highly reproducible [4,5,6,7,8,9,23,24]. However, the overall average will be low if only a few fields have a high TB. Additionally, if a high TB is focally found, the impact may be diluted by evaluating multiple fields [2]. Some researchers have reported results evaluated using immunohistochemistry to confirm TB [15,16]. This has the advantage of easier visualization of TBs. Indeed, it may be helpful to increase the reproducibility between researchers. However, not all pathology departments can perform immunohistochemistry, and its universality may therefore be limited. In addition, unless PTB is evaluated by performing immunohistochemistry on all tumor blocks, a limitation of representation is always encountered. In ITBCC guidelines, TB is evaluated on the basis of hematoxylin and eosin (H&E) [2]. This statement is a strong recommendation with moderate-quality evidence [2]. The evidence suggests that the prognostic impacts of PTB between H&E and immunohistochemistry does not differ materially [2]. The applied criteria for H&E staining and immunohistochemistry can differ. However, the information for the detailed criteria is unclear. Cumulative studies for evaluating PTB, including the counting location, applying stain, and scoring systems, are needed to determine the optimal method.

According to ITBCC recommendations, PTB is evaluated using a three-tier grading system as low budding (0–4 budding foci per a hotspot, area = 0.785 mm^2^), intermediatebudding (5–9 buddings per a hotspot, area = 0.785 mm^2^), and high budding (≥10 buddings per a hotspot, area = 0.785 mm^2^) [2]. However, the clinicopathological significance of intermediate budding remains controversial. Notably, interobserver discrepancies are frequently found in cases with the boundaries of each grade. Simple criteria may reduce interobserver variability in daily practice. In the present study, the clinicopathological impacts of high grade were mainly investigated. Therefore, we recommend a two-tiered system, instead of a three-tiered system. When analyzed using a three-tiered system, the results showed the clinicopathological and prognostic significance of PTB. CRCs with the same PTB grade can have different significance, depending on the tumor stage. Among the three grades of PTB, low and high grades have been reported to have better and worse prognoses, respectively. However, the prognostic role of intermediate budding may vary depending on the reporting. Previous reports have suggested that a pT1 CRC with an intermediate budding is associated with lymph node metastasis [17,25,26,27]. However, it has not been associated with recurrence or mortality in patients with Stage II CRC [28,29,30,31]. Therefore, the evaluation of a two-tier system may also be required for PTB. Roy et al. reported that there was no difference in recurrence-free survival between intermediate and high PTB grades [21]. However, other reports have shown no difference in survival between intermediate and low PTB grades [32]. It is necessary to consider the implications of heterogeneity in the intermediate PTB grade. In addition, this is an unavoidable part of the hotspot observation. The evaluation of PTB is based on a pathologist’s observation, not a quantitative measurement, and there is inevitable heterogeneity in the intermediate-grade group. To provide detailed information, it is recommended to record the absolute number of PTBs [2]. The comparison of three-tier vs. two-tier systems can be important.

Evaluating a single hotspot for PTB may be challenging in CRC, which exhibits various histological components. We evaluated and compared PTBs in CRCs with mucinous and micropapillary components. In this study, this was evaluated by dividing the cases according to the presence of mucinous and micropapillary components. Unlike previous reports [15,33], if mucinous and micropapillary components were present, they were classified and evaluated. PTB was significantly higher in CRCs without the mucinous component than in those with the mucinous component (*p* = 0.008). However, there was no significant correlation between PTB and the micropapillary pattern (*p* = 0.123). High PTB was significantly correlated with worse survival in CRC patients. However, in the subgroup analysis based on histological components, the prognostic implications of PTB were different. We previously reported that the prognostic implications could differ according to the presence of histological components [11]. Interestingly, the presence of a mucinous component had no impact on the prognosis based on the presence of PTB. CRCs with high PTB showed poor prognosis in CRCs with a microcapillary pattern but not in those without a micropapillary pattern. In daily practice, the mucinous and micropapillary components may be seen as playing a minor part. It may be helpful if the analysis is performed while bearing in mind that there is an association between histological CRC findings and the presence of PTB. Because the micropapillary pattern can be mistaken for TB, differentiation of this micropapillary pattern is necessary when evaluating PTB. Previously, the association of ITB with medullary, mucinous, and signet ring cell carcinomas has been reported [15]. In one study, 12 cases of mucinous carcinoma were analyzed, and high ITB was shown in 3 out of 12 of these cases. The patients with high ITB levels were found to have a poor prognosis. However, in their study, there was no information on PTB, as a study of ITB was conducted. Okuyama conducted an evaluation of TB in a 31-case mucinous carcinoma study [33]. TB is associated with venous invasion, lymph node metastases, distant metastases, and higher pTNM stages in mucinous carcinoma [33]. In addition, mucinous carcinoma with high TB had a poor prognosis [33].

Additionally, the concept of ITB was evaluated in CRCs. ITB may help us to understand the relevance of the intratumoral microenvironment. We previously studied CRCs, including histological components, metastatic lymph node ratio, and ITB, through a meta-analysis [34]. Our meta-analysis was performed in 2022 [34]. The number of eligible studies from 2017 to 2022 was 10 among 13 included studies [34]. Unfortunately, several studies have been published since the introduction of the ITBCC guidelines [2]. Future reviews may include ITB results and provide different recommendations. In our meta-analysis, the estimated rate of high ITB was 0.233 (95% confidence interval [CI] 0.177–0.299) [34]. When the criteria for high ITB were 5 and 10 ITBs, the estimated rates of high ITB were 0.321 (95% CI 0.175–0.512) and 0.222 (95% CI 0.149–0.317), respectively [34]. High ITB is associated with tumor grade, lymphatic invasion, perineural invasion, lymph node metastasis, and pT stage [34]. In addition, ITB was significantly higher in the medullary and signet ring cell subtypes than in the conventional and mucinous subtypes [34]. In the previous meta-analysis, the rates of high ITB were compared using the tumor regression grade of rectal cancers. Interestingly, rectal cancers with tumor regression grade 2–3 showed higher estimated rates of high ITB than those with tumor regression grade 0–1. ITB was significantly correlated with poor survival in CRC patients [34]. A further evaluation of the correlation between ITB and PTB in surgical specimens will be worthwhile.

## 5. Conclusions

Taken together, the findings indicate that high PTB is significantly correlated with aggressive tumor behavior, including lymphatic invasion, lymph node metastases, and distant metastases. In addition, CRC patients with high PTB exhibit worse recurrence-free survival than those with low PTB. However, in CRCs with a mucinous component, no significant difference in survival is found between patients with high and low PTB rates.

## Figures and Tables

**Table 1:** The correlation between the presence of peritumoral budding and clinicopathological parameters in colorectal cancers.

	Peritumoral Budding		*p*-Value
	High	Low	
Total (*n* = 266)	104 (39.1)	162 (60.9)	
Age (years)	63.45 ± 12.92	63.67 ± 12.94	0.892
Sex			
Male	51 (49.0)	84 (51.9)	0.707
Female	53 (51.0)	78 (48.1)	
Tumor size			
≤5 cm	37 (35.6)	69 (42.6)	0.305
>5 cm	67 (64.4)	93 (57.4)	
Tumor size (cm)	5.57 ± 1.85	5.39 ± 2.21	0.505
Location of tumor			
Right colon	41 (39.4)	87 (53.7)	***0.024***
Left colon	63 (60.6)	93 (46.3)	
Tumor differentiation			
Well or Moderately	79 (76.0)	132 (81.5)	0.282
Poorly	25 (24.0)	30 (18.5)	
Vascular invasion			
Present	11 (10.6)	13 (8.0)	0.515
Absent	93 (89.4)	149 (92.0)	
Lymphatic invasion			
Present	35 (33.7)	35 (21.6)	***0.033***
Absent	69 (66.3)	127 (78.4)	
Perineural invasion			
Present	23 (22.1)	21 (13.0)	0.063
Absent	81 (77.9)	141 (87.0)	
pT stage			
pT1-2	14 (13.5)	27 (16.7)	0.602
pT3-4	90 (86.5)	135 (83.3)	
Lymph node metastasis			
Present	67 (64.4)	79 (51.2)	***0.016***
Absent	37 (35.6)	83 (48.8)	
Metastatic lymph node ratio	0.16 ± 0.24	0.11 ± 0.21	0.087
Distant metastasis			
Present	18 (17.3)	11 (6.8)	***0.009***
Absent	86 (82.7)	151 (93.2)	
pTNM stage			
I-II	35 (33.7)	80 (49.4)	***0.016***
III-IV	69 (66.3)	82 (50.6)	

Numbers in parentheses represent percentages. *p* < 0.05 is highlighted in Italic bold.

**Table 2:** The correlation between peritumoral budding and histologic subtypes in colorectal cancers.

	Peritumoral Budding		*p*-Value
	High	Low	
Total (*n* = 266)	104 (39.1)	162 (60.9)	
Mucinous component			
Present	10 (9.6)	36 (22.2)	***0.008***
Absent	94 (90.4)	126 (77.8)	
Micropapillary component			
Present	23 (22.1)	51 (31.5)	0.123
Absent	81 (77.9)	111 (68.5)	

Numbers in parentheses represent percentages. *p* < 0.05 are highlighted in Italic bold.

## Data Availability

Data are contained within the article.
